# Evaluation of Coronal Soft Tissue Regeneration With Bovine Collagen Matrix Augmentation Over an Immediate Implant: A Randomized Controlled Trial

**DOI:** 10.7759/cureus.104550

**Published:** 2026-03-02

**Authors:** Dinesh Kumar Ravichandran, Karthikeyan G R, Balaguhan Balasubramanian, Deepak Velu, Veeramuthu M, Indrapriyadharshini Karthikeyan, Krishna Pragathy P

**Affiliations:** 1 Oral and Maxillofacial Surgery, Karpaga Vinayaga Institute of Dental Sciences, Chengalpet, IND; 2 Public Health Dentistry, Karpaga Vinayaga Institute of Dental Sciences, Chengalpet, IND

**Keywords:** collagen membrane, coronal augmentation, crestal soft tissue thickness, gingival tissue regeneration, immediate implant, soft tissue regeneration

## Abstract

Aim: This study aims to evaluate the use of augmented bovine-derived collagen to regenerate coronal soft tissue at the time of immediate implant placement in the aesthetic zone.

Materials and methods: Thirty-six individuals who underwent immediate implant placement from December 2024 to July 2025 were split at random into two groups. For Group I, a split-thickness flap was created, and a bovine collagen matrix was left coronally placed and sutured to the mucosa. For Group II, a full-thickness coronally repositioned flap was reflected and sutured to the mucosa. The clinical effectiveness of this collagen membrane, as measured by the volume of soft tissue (VST) regenerated and covered over the coronal surface of the implant, was evaluated by comparing the VST preoperatively after extraction (T0) and at the third postoperative month (T1) using an endodontic reamer and color map analysis. The effectiveness of collagen by means of epithelialization and granulation tissue formation at T1 was measured by performing a punch biopsy. Histologically, at T1, the nature of the regenerated soft tissue at the coronal part of the implant was compared with its native tissue.

Results: The clinical outcome results proved that Group I showed significant results with respect to VST, epithelialization, and granulation tissue formation. This difference shows that it is statistically significant (t=6.39; p=0.001), suggesting a better outcome in Group I than Group II. Histologically, Group I and Group II showed normal epithelization of their native tissue without any presence of inflammatory or granulation tissue.

Conclusion: The clinical outcome results proved that the bovine-derived collagen membrane showed significant results and could be used as an alternative to the coronally advanced flap.

## Introduction

Dental implant therapy is a reliable and successful method of restoring lost teeth. Several methods, including the flapless procedure, are suggested in light of the significance of maintaining bone volume and enhancing cosmetic outcomes [1]. Clinical studies have confirmed that the flap approach, involving total mucoperiosteal flap elevation, denudes buccal bone and promotes further resorption after tooth extraction [2]. Reduced vertical bone loss was observed with the flapless approach [1]. According to clinical data on dental implants, maintaining the integrity of the soft tissues may not require the loss of keratinized mucosa (KM) surrounding the implant and may not be associated with accelerated bone loss [3,4]. In contrast, a larger zone of KM, however, may be more advantageous for the long-term upkeep of dental implants and may better preserve the integrity of both soft and hard tissues, and the loss of KM may lead to soft tissue recession and poor oral hygiene [5-7]. This led to a clinical recommendation for the width of KM to be 2 mm, a size comparable to the keratinized gingiva zone that is advised to be sufficient around teeth [8-10]. Hence, the augmentative procedures used to increase the KM width include vestibuloplasty or an apically repositioned flap (ARF) [11]. Roll and pedicle flaps, free gingival grafts (FGG), and subepithelial connective grafts (SCTG) are among the exemplary methods [12-14]. ARF with or without the addition of autologous tissue grafts or other substitutes might be considered for the significant shrinkage of the augmented KM over time [15]. Autologous tissue grafts were used for their better predictability and stability [16]. Harvesting an autologous graft creates a second surgical site and may lead to untoward perioperative complications, and the size of the graft is limited by the presence of the greater palatine neurovascular bundle [17,18]. Connective tissue grafts (CTGs) are performed to increase the gingival thickness for root coverage procedures [19]. Enhancing the aesthetic outcome of dental implants is the main objective of soft tissue augmentation procedures. Abnormal labial or buccal placement of dental implants, healing or implant abutments after immediate implant insertion, and physiological soft tissue remodelling can all cause mucosal recession. The mucosal recession can be prevented by augmenting the peri-implant mucosa [20]. To prevent shimmering through implant components, particularly titanium, it may be necessary to increase the thickness of the peri-implant mucosa. CTGs are now the first-line treatment, especially in the aesthetic zone [21]. The "masking effect" of the abutment is generally agreed to require 2 mm of peri-implant mucosa [22]. The coronally advanced flap can disrupt papillary blood supply, which might lead to further gingival recession. Hence, by using the envelope flap, placement of the collagen matrix over the implant can provide better coronal healing. Hence, this study aimed to compare the formation of coronal soft tissue between the coronally advanced flap and collagen membrane.

## Materials and methods

A prospective, randomized controlled study was conducted at the Department of Oral and Maxillofacial Surgery, Karpaga Vinayaga Institute of Dental Sciences and Hospital, Chengalpet, India, from February 2025 to September 2025, after obtaining approval from the Institutional Ethics Committee of Karpaga Vinayaga Institute of Dental Sciences (approval number: KIDS/RAC/2024/V/024). It included 36 individuals who were randomly assigned into two groups: Group I (n=18) and Group II (n=18). This study was registered prospectively with the Clinical Trials Registry-India (CTRI) under registration number CTRI/2026/01/100873.

The inclusion criteria were as follows: (a) individuals who reported for the removal of fractured or non-salvageable anterior teeth (canine to canine) with adjacent dentition present and (b) absence of pregnancy, diabetes mellitus, and any systemic diseases making them unfit for the procedure; (c) no prior history of smoking, drug addiction, or medication; (d) intact buccal bone plate in the extraction socket (atraumatic extraction); (e) presence of sufficient bone; and (f) good oral health. Any tooth with a periapical lesion, periodontitis, or bone loss is excluded from the study. In compliance with the 1975 Helsinki Declaration, as revised in 2000, this study was carried out. After receiving a thorough explanation of the treatment protocols, the individuals were given at least one week to sign the informed consent form.

Sampling

**Figure 1:**
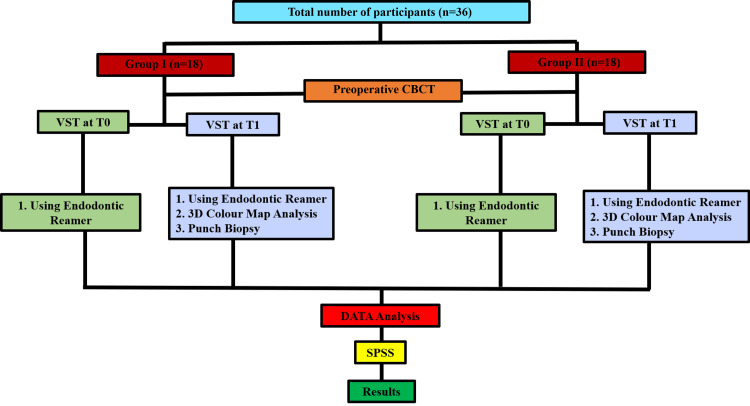
Methodology The flowchart describes the allocation, evaluation criteria, timeline, and procedure. CBCT: cone beam computed tomography; VST: volume of soft tissue; SPSS: Statistical Package for Social Sciences

Randomization was conducted using a computer-generated random sequence. Participants were assigned in a 1:1 ratio to the study and control groups. The randomization sequence was generated by an independent statistician who was not involved in participant recruitment or assessment. Allocation concealment was maintained using sequentially numbered, opaque, sealed envelopes, which were opened only after baseline data collection. Using the G*Power software (Version 3.1.9.7, Heinrich-Heine-Universität Düsseldorf, Düsseldorf, Germany), the sample size was calculated based on an 80% statistical power, a significance level of α=0.05, a 95% confidence interval, a 10% margin of error (E), and the mean soft tissue thickness reported in a previous study [23]. The estimated final sample size for the study was 36 participants (Figure 1).

Procedure

**Figure 2:**
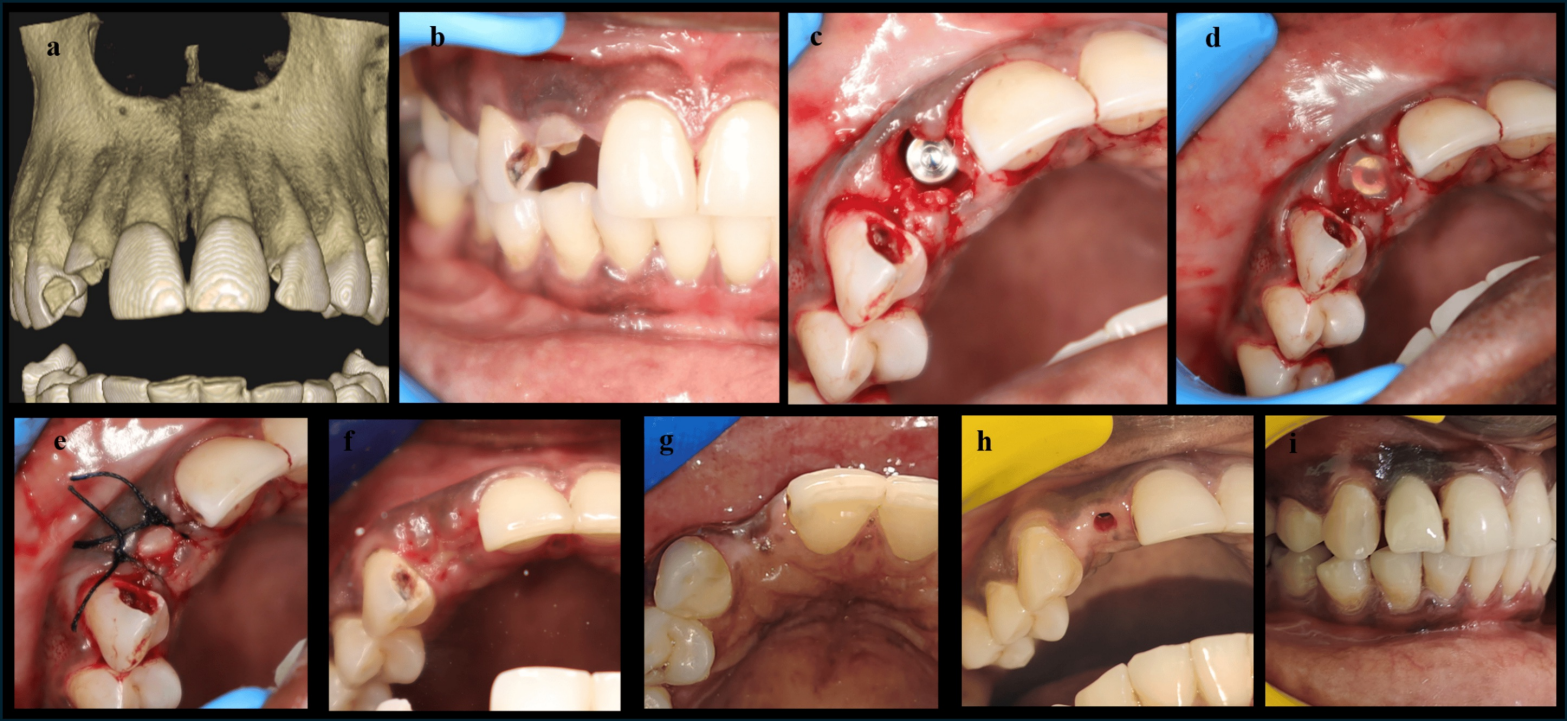
Procedure (a) Pre-op CBCT. (b) Tooth indicated 12. (c) Implant placement. (d) Collagen membrane augmentation. (e) Post-op. (f) Two weeks post-op. (g) Three months post-op. (h) Punch biopsy 12. (i) Final prosthesis. CBCT: cone beam computed tomography

At least six weeks prior to surgery, scaling was performed, and oral hygiene instructions were given. All the procedures were performed by a single surgeon. The indicated tooth extraction was carried out in an atraumatic manner. The usual osteotomy drills were used to perform osteotomy sequentially, and cylindrical implants with a minimum dimension of 3.3×10 mm were placed at 25 rpm and 35 Ncm torque (MIS, Dentsply Sirona, Charlotte, North Carolina, United States), using a one-stage surgical approach. No bone grafts were used in all cases. The collagen membrane was trimmed and hydrated in sterile saline solution for 10 minutes before securing. In Group I, an envelope split-thickness flap was created, and after the implant placement, the collagen membrane was positioned coronally, and the flap was secured with a simple interrupted suture using non-resorbable suture (Figure 2a-2g). In Group II, a crevicular incision with two vertical releasing incisions was placed, and a full-thickness mucoperiosteal flap was reflected. After implant placement, the flaps are repositioned coronally to cover the implant with mucosa and secured with non-resorbable sutures. All the implants were placed 1 mm below the crest. Postoperatively, individuals were instructed to rinse with 0.12% chlorhexidine mouthwash twice daily for two weeks. At the second postoperative week, sutures were removed. A soft diet was recommended post-surgery for four weeks. The augmented sites in both groups healed uneventfully, with no sign of inflammation or wound dehiscence. No case of peri-implantitis was reported postoperatively. All the implants showed successful osseointegration and were loaded according to the standard protocol after a healing period of three months. At the third postoperative month, individuals were reviewed, and healing abutments were positioned on the implants. While providing healing abutments, a punch biopsy was taken at the coronal region and sent for histopathological study. The impressions were taken, and fixed prostheses were provided (Figure 2h-2i).

Volume of soft tissue (VST) analysis

Using an Endodontic Reamer

**Figure 3:**
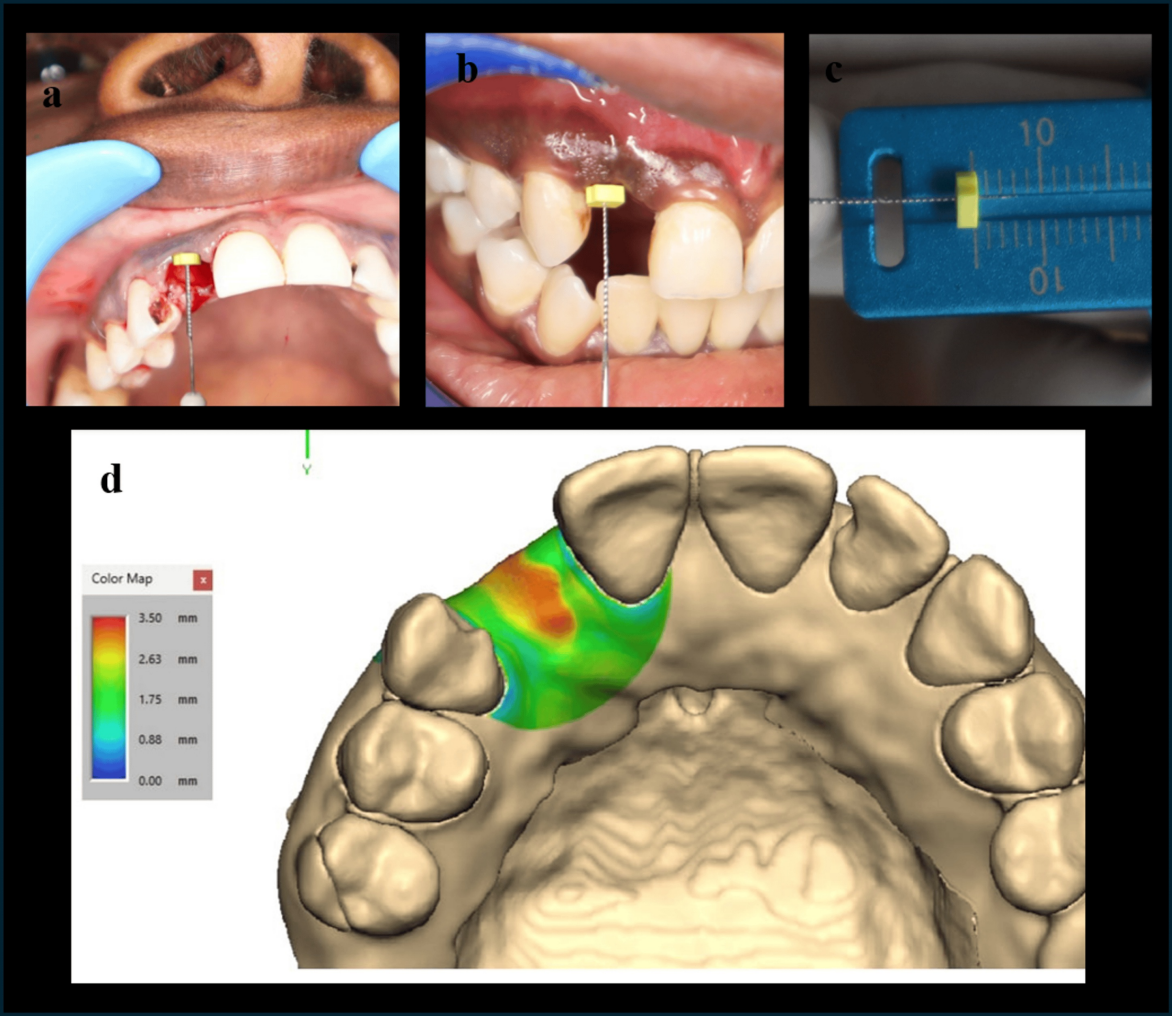
VST analysis (a) VST evaluation using an endodontic reamer at T0. (b) VST evaluation using an endodontic reamer at T1. (c) Calliper. (d) VST evaluation using a 3D color map analysis. VST: volume of soft tissue

Soft tissue thickness measurements were taken perioperatively after extraction (T0) and at the third postoperative month (T1). Using an endodontic reamer with a silicone disc stopper, it was penetrated through the marginal gingiva until the stopper hit the crestal bone. The stop was positioned in firm intimate contact with the surface of the mucosa. After the meticulous removal of the reamer with the stopper in position, penetration depth was measured using a calliper (Figure 3a-3c). In Group II at T0, the VST is measured at 2 mm below the marginal gingiva of the buccal and palatal mucosa, which are going to advance coronally to cover the implant site.

Using a 3D Color Map Analysis

The soft tissue dimensional analysis was evaluated using 3D deviation analysis. The cone beam computed tomography (CBCT) at T0 was taken to evaluate the bone at the implant site. At T1, an intraoral scan was done. The surface registration was done by superimposing the CBCT and intraoral scan to evaluate the augmented soft tissue thickness at the coronal region (Figure 3d). The evaluator was blinded, and the cases were randomized. The color map scale (on the left) ranges from 0.00 mm (blue) to 3.50 mm (red). The colors indicate that the surface deviates in relation to the implant region. Blue/green means smaller deviation/volume. Yellow/red means greater deviation/volume. The orange predominantly at the augmented site ranges from ~3±2 mm to ~2.2±3 mm in Groups I and II, respectively.

Statistical analysis

The data were compiled systematically in a Microsoft Excel spreadsheet (Microsoft Corporation, Redmond, Washington, United States) and subjected to statistical analysis using IBM SPSS Statistics for Windows, Version 27.0 (IBM Corp., Armonk, New York, United States). A paired t-test was used for intragroup comparison, and an unpaired t-test was used for intergroup comparison.

## Results

Table 1 describes the demographic details of the study participants. The mean age of participants in Group I and Group II was 37.89±6.52 and 36.72±8.09 years, respectively. The age range for Group I and Group II was 27-48 years and 25-54 years, respectively. In the maxilla, the implant sites were central incisor (eight cases; 44.4%), lateral incisor (five cases; 27.7%), and canine (one case; 5.5%) in Group I and central incisor (six cases; 33.3%), lateral incisor (seven cases; 38.8%), and canine (two cases; 11.1%) in Group II. In the mandible, Group I and Group II showed an equal distribution of implant sites in the central incisor (two cases; 11.1%), whereas the lateral incisor was slightly more in Group I (two cases; 11.1%) compared to Group II (one case; 5.5%). Overall, both groups demonstrated a comparable distribution of implant sites, suggesting a uniform sample composition.

**Table 1:** Demographic characteristics of the study participants Participant demographics are presented as mean±standard deviation (SD), along with the age range. The distribution of gender and implant-site locations in the maxilla and mandible are reported as frequencies (n).

Characteristics		Group I	Group II
Age (year)	Mean	37.89	36.72
	SD	6.52	8.09
	Range	27-48	25-54
Gender (n)	Male	10	12
	Female	8	6
Implant site (n)	Maxilla: central	8 (44.4%)	6 (33.3%)
	Maxilla: lateral	5 (27.7%)	7 (38.8%)
	Maxilla: canine	1 (5.5%)	2 (11.1%)
	Mandible: central	2 (11.1%)	2 (11.1%)
	Mandible: lateral	2 (11.1%)	1 (5.5%)
	Mandible: canine	0	0

Table 2 presents the results of the Shapiro-Wilk normality test for VST measurements assessed using the endodontic reamer method as well as the 3D color map analysis. 

**Table 2:** Normality test for VST evaluation using an endodontic reamer and using 3D color map comparison The data were assessed for normality by using the Shapiro-Wilk test. The data were normally distributed; therefore, a parametric test was used. VST: volume of soft tissue

Variables	Timeline	Groups	Statistic	df	Sig.
VST evaluation using an endodontic reamer	Baseline	Group 1	0.969	18	0.786
		Group 2	0.2	18	0.885
	3 months	Group 1	0.864	18	0.014
		Group 2	0.907	18	0.075
VST evaluation using 3D color map comparison	-	Group 1	0.88	18	0.026
	-	Group 2	0.921	18	0.133

In Table 3, the intragroup comparison of VST using an endodontic reamer between T0 and T1 found that in Group I, there was a statistically significant difference with a t-value of -16.595 and a p-value of 0.000 (p<0.05). Group II is also statistically significant, with a t-value of -3.217 and a p-value of 0.005 (p<0.05). This significance suggested that in both groups, there was adequate regeneration of coronal soft tissue between T0 and T1.

**Table 3:** Intragroup VST comparison VST evaluation using an endodontic reamer, between T0 and T1. A paired t-test was applied (*p<0.05 indicates statistically significant). VST: volume of soft tissue

Group	Period	Mean	SD	t-value	P-value
Group I	T0	2.6444	0.29	-16.595	0.000*
	T1	3.2444	0.25257		
Group II	T0	2.6222	0.2102	-3.217	0.005*
	T1	2.7278	0.23214		

In Table 4, the intergroup comparison at T0 was not statistically significant (t=0.262; p=0.795) among the groups, indicating that both groups were almost equal before the intervention. At T1, Group I showed a mean score of 3.24±0.25, while Group II had a mean score of 2.73±0.23. This difference shows that it is statistically significant (t=6.39; p=0.001), suggesting a better outcome in Group I than Group II.

**Table 4:** Intergroup VST comparison VST evaluation using an endodontic reamer, between T0 and T1. An unpaired t-test was applied (*p<0.05 indicates statistically significant). VST: volume of soft tissue

Period	Group	Mean	SD	t-value	P-value
T0	Group I	2.6444	0.29149	0.262	0.795
	Group II	2.6222	0.2102		
T1	Group I	3.2444	0.25257	6.39	0.001*
	Group II	2.7278	0.23214		

In Table 5, the mean VST by virtual color map comparison with CBCT revealed that 3.24±0.22 mm in Group I and 2.84±0.23 mm in Group II indicated a statistically significant difference between the two groups (t=5.322; p=0.001). Group I exhibited greater coronal soft tissue regeneration over the implant compared to Group II.

**Table 5:** VST analysis VST evaluation using 3D color map comparison at T1 between Group I and Group II. Independent t-tests were applied (*p<0.05 indicates statistically significant). VST: volume of soft tissue

Group	Mean	SD	t-value	P-value
Group I	3.2444	0.22022	5.322	0.001*
Group II	2.8444	0.23066		

Histological analysis

**Figure 4:**
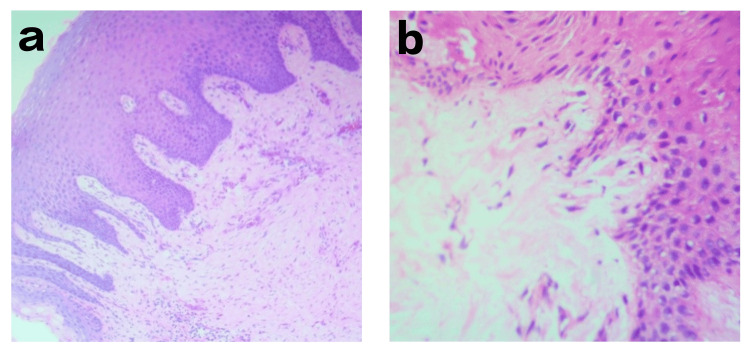
Histological analysis The histological study shows reduced inflammatory cells and granulation tissue replaced by collagen.

The punch biopsy was performed at the crest of the augmented site in both groups at the third postoperative month and was microscopically studied. The biopsy samples of both Group I and Group II showed normal keratinized gingival epithelium with fibrous collagenous structure. No signs of inflammatory cells and granulation tissue were present. The collagen has replaced the granulation tissue (Figure 4a-4b).

## Discussion

The peri-implant soft tissue is measured horizontally at the base of the peri-implant sulcus or at the most coronal part of the implant shoulder [19]. This horizontal measurement, defined in the past as "mid-facial peri-implant mucosa", has been used for the aesthetic evaluation around dental implants [22].

The coronal advanced flap (CAF), a workhorse flap for the closure of an immediate implant, can cause loss of vestibular depth and soft tissue tension, relocation of the mucogingival junction, and reduced vestibular depth, as mentioned by Buser et al. [24]. To overcome this drawback, in this study, Group I patients were operated on with a flapless technique to avoid releasing incisions, to avoid soft tissue complications, and also to improve the aesthetic outcome by maintaining the integrity of the underlying periosteal bed without compromising vascularity [25].

In this study, bovine collagen was used as a grafting material for peri-implant soft tissue augmentation as a soft tissue cover at the time of immediate implant placement in the anterior tooth region. Wiesner et al. studied the soft tissue thickness with or without augmentation of CTG harvested from the palate and found that there was statistically higher soft tissue thickness in the augmented group than the control group [26]. Regarding the usage of xenogenic collagen matrix (XCM) ahead of CTGs for the augmentation of buccal soft tissue, Cairo et al. found that even though CTG was effective, it was associated with longer chair time and greater morbidity, while the XCM was associated with shorter surgical time, lower postoperative morbidity, fewer anti-inflammatory reactions, and higher patient satisfaction [27].

In the present study, the mean value of VST in Group I was 3.24±0.22 mm, which was statistically significant (p=0.001) when compared with Group II (2.84±0.23 mm). This result might be in contrast to the study to find the influence of gingival tissue thickness on crestal bone loss around dental implants conducted by Linkevicius et al., who concluded that less than 2 mm of peri-implant mucosal loss has been associated with marginal bone loss up to 1.45 mm at the end of one year [28]. The higher the thickness of the regenerated volume of the coronal soft tissue, the less the crestal bone loss.

In this study, the VST of the regenerated coronal tissue over the implant was measured from the crest of the coronal mucosa to the crest of the alveolar bone by using an endodontic reamer and a gauge, whereas Zafiropoulos and John employed an endodontic reamer coupled with a metallic stent for the same [23]. Byun et al. analyzed the soft tissue changes by 3D superimposition color map analysis [29]. Both the endodontic reamer measures and the 3D color map superimposition proved that Group I attained a significant increase in the volume of coronal soft tissue regeneration over the implant surface compared to Group II.

Histologically, at T1, there was no significant difference found among the groups, which might be in accordance with the study conducted by Zang et al.; by using acellular dermal matrix for peri-implant vertical soft tissue augmentation, the histological and immunohistochemical outcomes showed no differences between the study groups [30].

Clinical implications

Collagen matrix augmentation during immediate implant placement in the aesthetic zone enhances soft tissue thickness, maintains vestibular architecture, shows good histologic integration, and may serve as a predictable alternative to CTGs.

Limitations

Though there were limitations like a smaller sample size, a minimum period (three months) of follow-up, and all the cases belonging to single tooth replacement, the collagen membrane showed better results ahead of all other repositioning flaps and CTGs for soft tissue augmentation around dental implants.

Future recommendations

Further long-term, multicenter studies with larger sample sizes are recommended to evaluate the stability of augmented peri-implant soft tissue. A direct comparison of autogenous CTGs, bovine collagen matrix, and other xenogenic or allogenic soft tissue substitutes would aid in the development of definitive clinical guidelines.

## Conclusions

The clinical outcome results proved that the bovine-derived collagen membrane showed significant results with respect to the volume of soft tissue and epithelialization without any histological changes of its native soft tissue while maintaining the vestibular architecture. The flapless approach helped preserve the periosteal blood supply, avoided releasing incisions, and maintained the integrity of the mucogingival complex, thereby minimizing soft tissue complications and enhancing aesthetic outcomes in the anterior region. Its usage as a substitute for connective tissue grafts offers adequate tissue thickness with reduced surgical morbidity and improved patient comfort. Furthermore, increased peri-implant soft tissue thickness may contribute to reduced crestal bone loss and improved long-term peri-implant stability. Given the constraints of this study, bovine collagen seems to serve as an alternative to CTGs for soft tissue augmentation around dental implants in immediate implant placement at aesthetic zones.
